# Efficient Biosafe Inactivation of Classical Swine Fever Virus While Preserving Viral RNA for Molecular Diagnosis

**DOI:** 10.3390/v18080885

**Published:** 2026-08-12

**Authors:** Adriana Muñoz-Aguilera, Aníbal Asilvera, Sara Puente-Marín, Xavier Abad, Cristina Riquelme, Saray Heredia, Yoandry Hinojosa, Liani Coronado, Christopher Helm, Jerry Torrison, Llilianne Ganges

**Affiliations:** 1 WOAH Reference Laboratory for Classical Swine Fever, IRTA-CReSA, 08193 Barcelona, Spain; adriana.munoz@irta.cat (A.M.-A.); anibal.asilvera@irta.cat (A.A.); sara.puente@irta.cat (S.P.-M.); xavier.abad@irta.cat (X.A.); cristina.riquelme@irta.cat (C.R.); saray.heredia@irta.cat (S.H.); yoandry.hinojosa@irta.cat (Y.H.); liani.coronado@irta.cat (L.C.); 2 Unitat Mixta d’Investigació, IRTA-UAB en Sanitat Animal, Centre de Recerca en Sanitat Animal (CReSA), Bellaterra, 08193 Barcelona, Spain; 3 IRTA, Programa de Sanitat Animal, Centre de Recerca en Sanitat Animal (CReSA), Bellaterra, 08193 Barcelona, Spain; 4 Subgerencia de Análisis y Diagnóstico, Instituto Colombiano Agropecuario (ICA), Bogot 110911, Colombia; 5 Longhorn Vaccines and Diagnostics, LLC, Bethesda, MD 20814, USA; chris@lhnvd.com (C.H.); jerryt@lhnvd.com (J.T.)

**Keywords:** classical swine fever virus, PrimeStore^®^ MTM, virus inactivation, RT-qPCR, RNA stability, molecular diagnostics, diagnostic harmonization

## Abstract

Classical swine fever (CSF) is a highly contagious transboundary animal disease that causes major economic losses to the global swine industry and remains notifiable to the World Organisation for Animal Health (WOAH). Handling and transport of infectious classical swine fever virus (CSFV)-positive samples require biosafety level 3 (BSL-3) containment, limiting diagnostic capacity and interlaboratory exchange in many regions. In this study, we evaluated the efficacy of PrimeStore^®^ Molecular Transport Medium (PS-MTM) for eliminating detectable CSFV infectivity while preserving viral ribonucleic acid (RNA) detectability for downstream molecular detection. Validation assays were performed using the CSFV Alfort/187 reference strain and clinical samples collected from pigs experimentally infected with the Catalonia01 and Margarita CSFV strains. Residual infectivity was assessed by virus isolation, whereas viral RNA detection and stability were evaluated using the WOAH-recommended real-time reverse transcription quantitative polymerase chain reaction(RT-qPCR) assay. Following PS-MTM treatment, no residual infectivity was detected in either cell culture-derived virus stocks or clinical samples, including extensive replicate testing and three subsequent blind passages. RT-qPCR analyses confirmed preservation and stability of detectable CSFV RNA for at least 60 days under the storage conditions evaluated, including refrigerated storage and room temperature storage for Catalonia01-derived samples. Overall, these findings demonstrate that PS-MTM eliminates detectable CSFV infectivity while preserving viral RNA for molecular diagnosis. The implementation of PS-MTM may facilitate safer handling, transport, and interlaboratory exchange of CSFV-positive samples, contributing to improved biosafety, diagnostic harmonization, and global surveillance capacity for this transboundary animal disease.

## 1. Introduction

Classical swine fever (CSF) remains one of the most important transboundary animal diseases affecting domestic pigs and wild boar populations worldwide, causing severe economic losses to the global swine industry. The disease is notifiable to the World Organisation for Animal Health (WOAH), and outbreaks continue to represent a major threat to animal health, food security, and international trade [1,2]. The causative agent, CSF virus (CSFV), is an enveloped positive-sense single-stranded ribonucleic acid (RNA) virus belonging to the genus *Pestivirus* within the family *Flaviviridae*. Owing to its high transmissibility and capacity for rapid dissemination through animal movements and trade, CSFV remains a major barrier to international commerce involving swine and swine-derived products. Consequently, rapid diagnostic capacity, harmonized laboratory procedures, and safe handling of infectious material are essential components of national and international CSF surveillance and control programs [3].

CSFV is classified as a risk group 3 pathogen and therefore requires manipulation under biosafety level 3 (BSL-3) containment conditions. However, access to BSL-3 facilities remains limited in many regions due to the substantial financial, infrastructural, and logistical requirements associated with their operation. Consequently, many veterinary diagnostic laboratories, particularly in endemic or resource-limited settings, face difficulties in performing standardized molecular diagnostics, participating in proficiency testing exercises, exchanging reference materials, and implementing harmonized diagnostic procedures according to WOAH standards [3]. In addition, shipment of potentially infectious samples requires strict biosafety regulations, specialized packaging systems, cold-chain maintenance, and regulatory oversight, which further complicate large-scale surveillance activities and international interlaboratory collaborations. Several physical and chemical approaches for CSFV inactivation have previously been evaluated, including ultraviolet irradiation [4], hydrostatic pressure [5], high-temperature treatment [6], spray-drying [7], and salt-based inactivation systems in biological matrices [8,9]. These studies demonstrated that CSFV infectivity can be substantially reduced while maintaining the integrity of viral RNA or antigenic components required for downstream molecular or serological analyses [7,10,11]. Nevertheless, many of these approaches are difficult to standardize across different sample types, require specialized equipment, or are not readily adaptable to routine diagnostic workflows and field conditions. Despite the availability of these approaches, few studies have specifically addressed the preparation of non-infectious CSFV-positive material suitable for safe transport, long-term storage, interlaboratory exercises, or international proficiency testing programs [10,12,13]. Previous studies demonstrated that chaotropic reagents such as Trizol^®^ can effectively abolish CSFV infectivity while preserving viral RNA for molecular analyses and epidemiological investigations [13], whereas validated approaches have also been described for the inactivation of CSFV-positive sera while maintaining antibody detectability for serological applications [10]. Nevertheless, the availability of standardized systems specifically designed for the biosafe transport and molecular diagnosis of inactivated CSFV samples remains limited. Reliable inactivation strategies capable of eliminating viral infectivity while preserving nucleic acid integrity therefore represent an important unmet need for CSFV diagnostics and surveillance. Such approaches would facilitate the distribution of reference materials, improve access to molecular testing in laboratories lacking BSL-3 infrastructure, and support the harmonization of diagnostic procedures. This need has become increasingly relevant following recent WOAH-coordinated interlaboratory evaluations, which emphasized the importance of standardized and biosafe transport of CSFV diagnostic materials.

PrimeStore^®^ Molecular Transport Medium (PS-MTM) is a Class II microbial nucleic acid storage and stabilization device that offers a promising solution to address these challenges. PS-MTM contains guanidinium thiocyanate as a chaotropic agent capable of lysing viral particles, inactivating nucleases, and stabilizing nucleic acids for downstream molecular applications [14,15]. Unlike Trizol^®^, which was primarily developed as a laboratory reagent for RNA extraction, PS-MTM was specifically designed as a specimen collection and transport medium that immediately inactivates infectious pathogens while stabilizing nucleic acids within the original clinical sample, thereby enabling biosafe transport prior to downstream molecular analysis [14]. Previous studies have validated PS-MTM for the safe collection, transport, storage, and molecular detection of several pathogens relevant to veterinary and public health [14,16,17,18]. In particular, Spruill-Harrell et al. [14] demonstrated complete loss of infectivity together with preservation of detectable viral nucleic acids following PS-MTM treatment for multiple high-consequence human pathogens. However, despite its increasing implementation in molecular diagnostic workflows, the performance of PS-MTM for CSFV inactivation and viral RNA preservation in diagnostically relevant matrices, including sera and swab samples, has not yet been evaluated, nor has the absence of residual infectivity following PS-MTM treatment been validated for this pathogen.

Therefore, the aim of the present study was to evaluate the efficacy of PS-MTM for eliminating detectable CSFV infectivity in both cell culture-derived virus stocks and clinical samples while preserving viral RNA detectability for downstream real-time reverse transcription quantitative polymerase chain reaction (RT-qPCR) analysis. By establishing a validated workflow for biosafe sample handling, transport, and molecular analysis, this study may contribute to improved diagnostic preparedness, interlaboratory exchange of reference materials, and harmonization of CSFV molecular diagnostics in accordance with WOAH recommendations.

## 2. Materials and Methods

### 2.1. Cells and Viruses

Viral production and titration assays were performed using the porcine kidney cell line (PK-15) (CCL-33; American Type Culture Collection [ATCC], Manassas, VA, USA), cultured in Eagle’s minimum essential medium supplemented with 5% fetal bovine serum (FBS). The CSFV Alfort/187 strain (genotype 1.1), kindly provided by the CSFV European Union Reference Laboratory (EURL), Hanover, Germany, was used for analytical inactivation assays employing cell culture-derived virus stocks. The CSFV Catalonia01 (genotype 2.3) and Margarita (genotype 1.4) strains were used for in vivo experiments. Viral stocks were titrated by endpoint dilution assay in 96-well tissue culture plates using the peroxidase-linked assay (PLA) [19]. The virus titers were calculated as 50% tissue culture infectious doses (TCID_50_)/mL [20].

### 2.2. CSFV Samples Used for PS-MTM Evaluation

The samples analyzed in the present study were obtained from two independent experimental CSFV infection studies previously conducted at IRTA-CReSA. No experimental animal infections were performed as part of the present work. Instead, previously collected samples were selected to evaluate the efficacy of PS-MTM for CSFV infectivity inactivation and the preservation of viral RNA detectability following treatment.

For the evaluation of CSFV inactivation under diagnostically relevant conditions, previously collected serum and nasal swab samples were selected from an independent study involving piglets experimentally infected with the moderately virulent CSFV Catalonia01 strain. Samples collected at 14 days post-infection (dpi) were selected for the present study based on their viral RNA loads and infectivity titers. The original animal experiment was approved by the Animal Experimentation Committee of the Generalitat of Catalonia (Approval No. 11970, 12 January 2023) and is described in a separate study currently in preparation.

Samples used to evaluate CSFV RNA detectability during storage following PS-MTM treatment were obtained from a previously published experimental infection study involving the highly virulent CSFV Margarita strain (genotype 1.4) [21]. In that study, serum, nasal, buccal, and rectal swab samples collected at 8 and 14 dpi were subsequently stored and used in the present work to assess the preservation of CSFV RNA detectability under refrigerated and room-temperature storage conditions following PS-MTM treatment. The original animal experiment was approved by the Animal Experimentation Committee of the Generalitat of Catalonia (Approval No. 12497, 7 October 2024).

All procedures involving infectious CSFV performed as part of the present study, including sample handling, PS-MTM treatment, and virus isolation assays, were conducted under biosafety level 3 (BSL-3) containment conditions at IRTA-CReSA (Bellaterra, Spain). The experimental animal studies from which the samples originated were conducted previously in accordance with Directive 2010/63/EU [22] on the protection of animals used for scientific purposes and under the ethical approvals indicated above.

### 2.3. CSFV Inactivation Using PS-MTM

**Figure 1:**
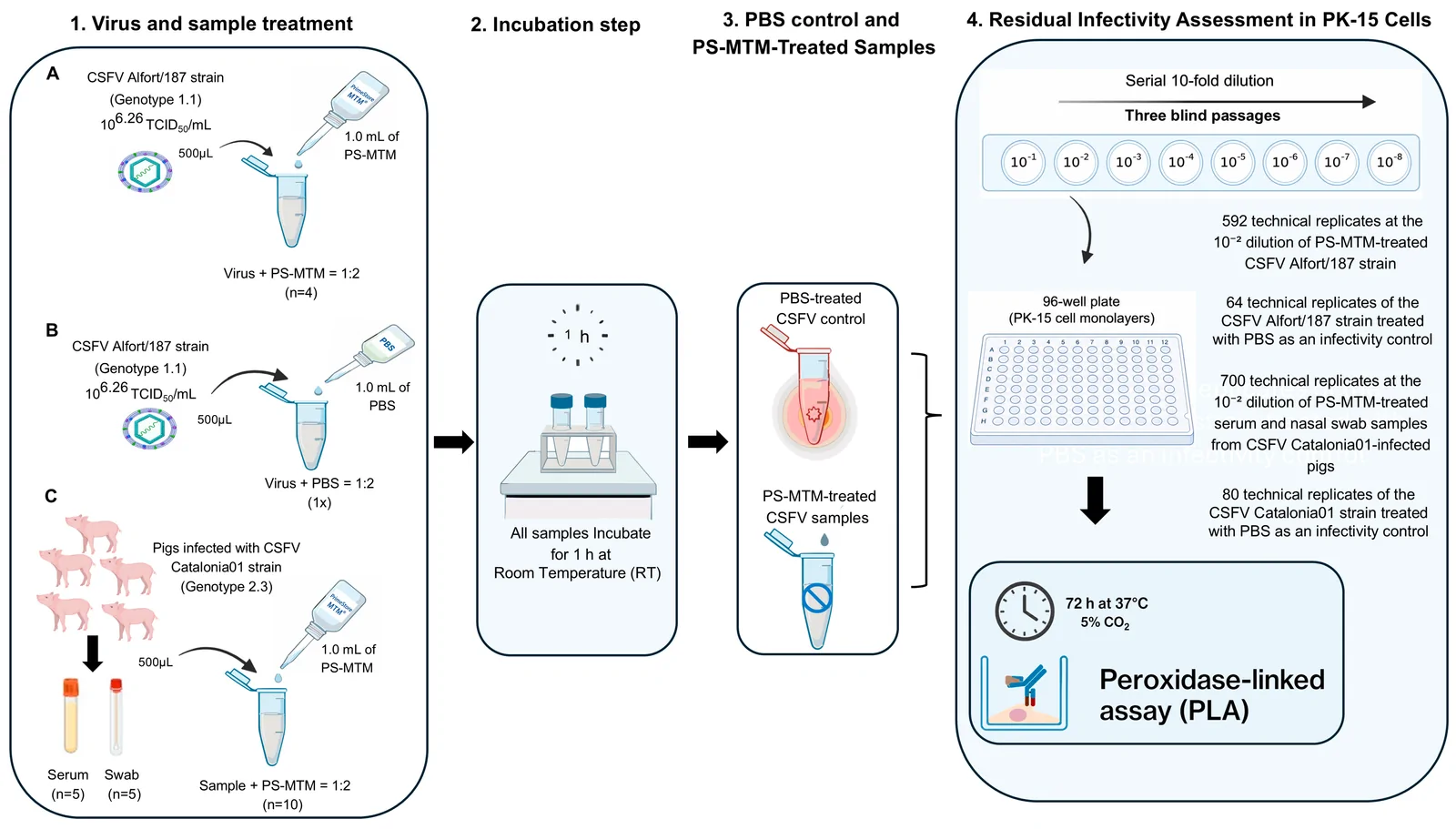
**Experimental workflow for the evaluation of CSFV inactivation using PS-MTM**. Cell culture-derived CSFV Alfort/187 and clinical serum and nasal swab samples from Catalonia01-infected pigs were treated with PS-MTM and assessed for residual infectivity in PK-15 cells. PBS-treated samples were included as infectivity controls. Abbreviations: CSFV, classical swine fever virus; PS-MTM, PrimeStore^®^ Molecular Transport Medium; PK-15, porcine kidney-15 cell line; PBS, phosphate-buffered saline; TCID_50_, 50% tissue culture infectious dose.

To evaluate the efficacy of PS-MTM (Longhorn Vaccines and Diagnostics, Bethesda, MD, USA) for CSFV inactivation, infectious materials were treated according to the manufacturer’s recommendations using a final sample-to-reagent ratio of 1:2. Initial analytical inactivation assays were performed using the CSFV Alfort/187 reference strain (genotype 1.1), with a previously determined viral titer of 10^6.26^ TCID_50_/mL. Briefly, 500 μL of infectious virus suspension were mixed with 1 mL of PS-MTM and incubated for 1 h at room temperature (RT; 22 ± 2 °C). PS-MTM-treated samples and the corresponding phosphate-buffered saline (PBS)-treated samples were processed in parallel, with the latter included as positive infectivity controls. To further evaluate the performance of PS-MTM in diagnostically relevant clinical matrices, serum and swab samples obtained from pigs experimentally infected with the CSFV Catalonia01 and Margarita strains were subjected to the same treatment protocol. Serum and nasal swab samples from Catalonia01-infected pigs were used both for infectivity validation and for subsequent assessment of CSFV RNA detectability during storage, whereas serum, nasal, buccal, and rectal swab samples from Margarita-infected pigs were used exclusively for the molecular assessment. Clinical specimens were directly mixed with PS-MTM without further processing. Briefly, 500 μL of each clinical specimen were mixed with 1 mL of PS-MTM and incubated for 1 h at RT prior to downstream analyses (Figure 1).

### 2.4. Validation of CSFV Inactivation

Residual infectivity following PS-MTM treatment was evaluated by virus isolation in PK-15 cells. Briefly, PS-MTM-treated samples and the corresponding PBS-treated controls were serially diluted tenfold and inoculated onto PK-15 cell monolayers seeded in 96-well plates. Following incubation for 72 h at 37 °C under 5% CO_2_, viral replication was assessed using the peroxidase-linked assay (PLA) as previously described by Terpstra et al. [19]. Viral titers were expressed as TCID_50_/mL and calculated according to the Reed-Muench method [20]. Absence of residual infectivity was defined as the lack of detectable viral replication following inoculation of PS-MTM-treated material onto PK-15 cells and three subsequent blind passages. PBS-treated CSFV Alfort/187 and Catalonia01 clinical-sample controls were included in parallel as positive infectivity controls to confirm the presence of infectious virus and the sensitivity of the virus isolation assay. To ensure robust validation of the inactivation protocol, a preliminary cytotoxicity assessment was performed to identify the most concentrated dilution of PS-MTM-treated material compatible with reliable interpretation in PK-15 cells. Based on this assessment, extensive technical replicate testing for residual infectivity was conducted at the 10^−2^ dilution. For the analytical validation assays using the CSFV Alfort/187 reference strain, four independent PS-MTM treatment preparations were performed, yielding a total of 592 technical replicates. For clinical validation, one serum sample and one nasal swab collected from each of five pigs experimentally infected with the CSFV Catalonia01 strain were independently treated with PS-MTM, resulting in 10 independently treated clinical specimens. Technical replicates consisted of independent virus isolation wells inoculated with aliquots of each PS-MTM-treated specimen at the selected 10^−2^ dilution, yielding a total of 700 technical replicates (Figure 1).

### 2.5. Assessment of CSFV RNA Detectability and Stability by RT-qPCR

RT-qPCR analyses were performed to evaluate the preservation of CSFV RNA detectability following PS-MTM treatment and during subsequent storage. For the Alfort/187 reference strain, the original viral preparation was analyzed before treatment. Following the 1 h incubation with PS-MTM, the four independently treated preparations were pooled into a single sample. CSFV RNA detectability was subsequently assessed in the pooled PS-MTM-treated material and in its corresponding 10^−2^ dilution.

**Figure 2:**
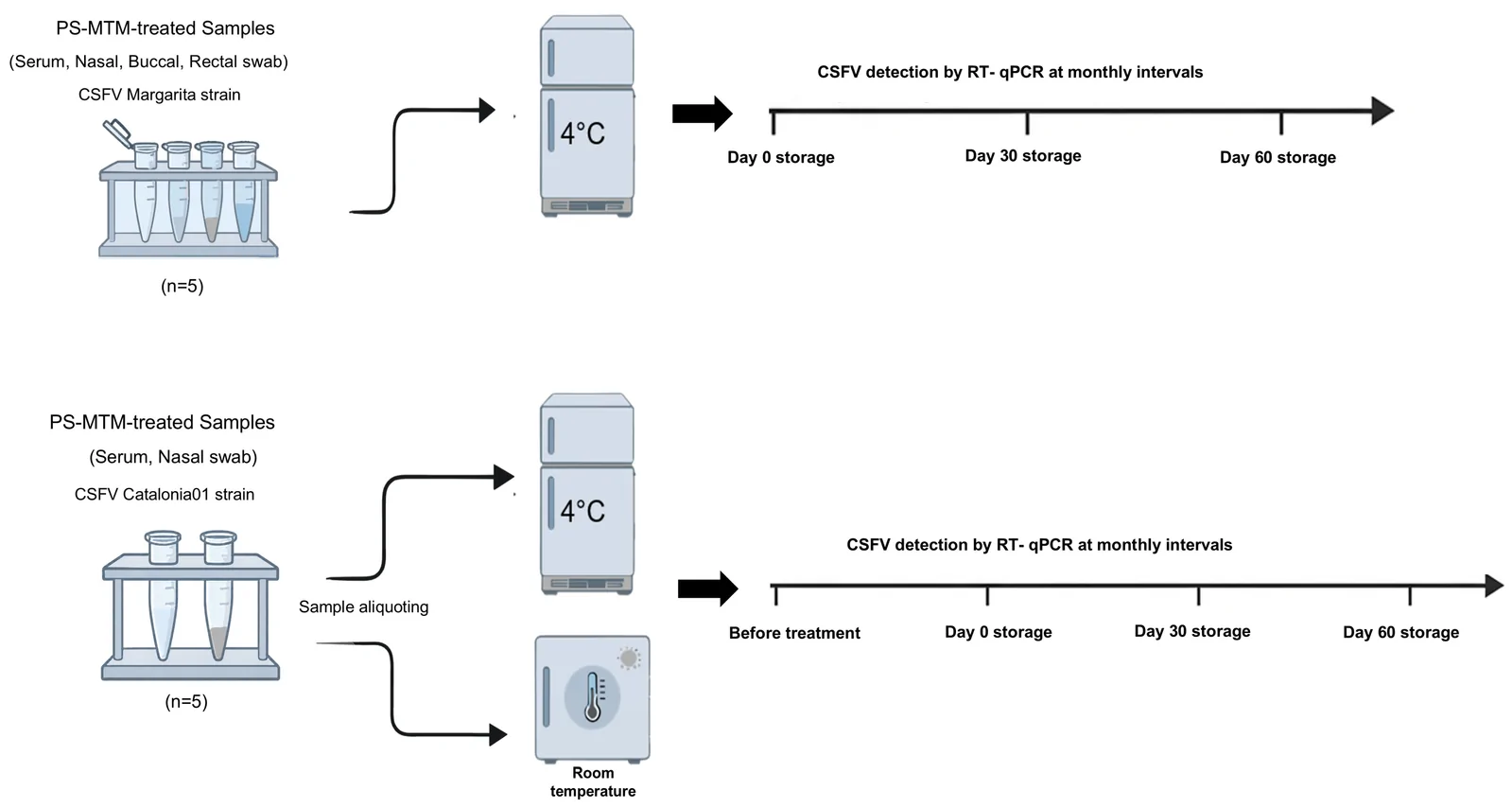
**Workflow for CSFV RNA stability assessment in PS-MTM-treated clinical samples.** Serum, nasal, buccal, and rectal swab samples from pigs experimentally infected with the CSFV Margarita strain were stored at 4 °C and analyzed by RT-qPCR at day 0, day 30, and day 60 after PS-MTM treatment. Serum and nasal swab samples from pigs experimentally infected with the CSFV Catalonia01 strain were aliquoted after PS-MTM treatment, stored at 4 °C and room temperature, and analyzed by RT-qPCR before treatment, at day 0, day 30, and day 60. The workflow was designed to assess the preservation and detectability of CSFV RNA during post-treatment storage. Abbreviations: RNA, ribonucleic acid; RT-qPCR, real-time reverse transcription quantitative polymerase chain reaction.

Long-term RNA stability analyses were performed using PS-MTM-treated serum, nasal, buccal, and rectal swab samples obtained from pigs experimentally infected with the CSFV Margarita strain. Samples were stored at 4 °C for 30 and 60 days prior to RNA extraction and RT-qPCR analysis (Figure 2). To further evaluate RNA stability under conditions representative of routine diagnostic shipment and storage, PS-MTM-treated serum and nasal swab samples obtained from pigs infected with the CSFV Catalonia01 strain were stored both under refrigerated conditions (4 °C) and at room temperature (22 ± 2 °C) for 30 and 60 days prior to molecular analysis (Figure 2).

CSFV RNA was extracted from untreated and PS-MTM-treated samples using the MagAttract 96 Cador Pathogen Kit (Qiagen, Hilden, Germany), following the manufacturer’s instructions from an initial sample volume of 200 µL. Following extraction, RNA samples were stored at −80 °C until analysis. Detection of CSFV RNA was performed using the WOAH-recommended pan-CSFV RT-qPCR assay previously described by Hoffmann et al. [23]. Reactions were performed in a final volume of 20 µL using AgPath-ID™ One-Step RT-PCR Reagents (Applied Biosystems, Waltham, MA, USA). Each clinical specimen was analyzed as an independent biological sample, and RT-qPCR was performed as a single determination at each sampling time. Samples were considered positive when cycle threshold (Ct) values were ≤40, whereas samples showing no detectable fluorescence signal were considered negative.

## 3. Results

### 3.1. PS-MTM Treatment Eliminated Detectable CSFV Infectivity in Cell Culture-Derived Virus and Clinical Samples

The capacity of PS-MTM to eliminate detectable CSFV infectivity was first evaluated using the cell culture-derived Alfort/187 reference strain. Before treatment, the viral stock had an infectious titer of 10^6.26^ TCID_50_/mL. Four independent PS-MTM treatment preparations were performed. A preliminary cytotoxicity assessment showed that the 10^−1^ dilution interfered with the interpretation of PK-15 cell monolayers, whereas no cytotoxicity affecting infectivity assessment was observed at the 10^−2^ dilution. Consequently, residual infectivity testing was performed at the 10^−2^ dilution.

**Figure 3:**
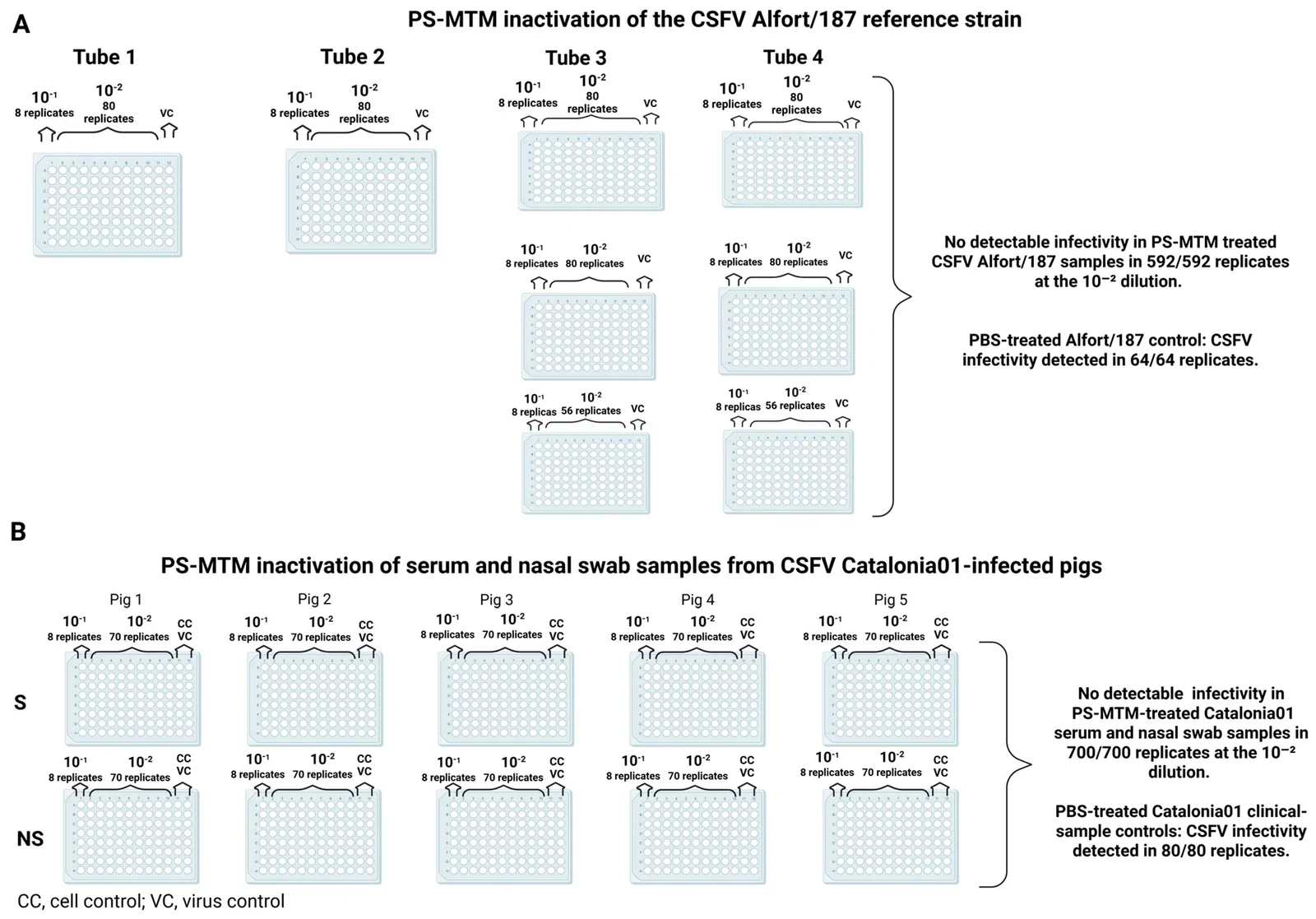
**Assessment of residual CSFV infectivity following PS-MTM treatment**. (**A**) Four independently treated preparations of the CSFV Alfort/187 reference strain were evaluated in PK-15 cells at the 10^−2^ dilution, together with the corresponding PBS-treated infectivity control. (**B**) Serum and nasal swab samples collected from five pigs experimentally infected with the CSFV Catalonia01 strain were independently treated with PS-MTM and evaluated under the same conditions. Viral replication was assessed using the peroxidase-linked assay after three blind passages. Numbers indicate the technical replicates evaluated. Abbreviations: S, serum; NS, nasal swab; CC, cell control; VC, virus control.

No viral replication was detected in any of the 592 technical replicates evaluated following PS-MTM treatment. Moreover, no detectable viral replication was observed after three subsequent blind passages. In contrast, viral replication was detected in all 64 technical replicates of the corresponding PBS-treated Alfort/187 infectivity control, confirming the infectivity of the starting material and the ability of the virus isolation procedure to detect infectious CSFV under the assay conditions used (Figure 3A).

The performance of PS-MTM was subsequently evaluated in diagnostically relevant clinical matrices. One serum sample and one nasal swab collected from each of five pigs experimentally infected with the moderately virulent CSFV Catalonia01 strain were independently treated with PS-MTM, resulting in 10 independently treated clinical samples. Before treatment, serum samples showed infectious titers ranging from approximately 10^6.5^ to 10^7^ TCID_50_/mL, whereas nasal swabs presented lower titers ranging from 10^2.2^ to 10^3.4^ TCID_50_/mL. No detectable viral replication was observed in any of the 700 technical replicates evaluated from the PS-MTM-treated serum and nasal swab samples at the 10^−2^ dilution. Viral replication also remained undetectable after three subsequent blind passages. By contrast, CSFV infectivity was detected in all 80 technical replicates of the corresponding PBS-treated clinical-sample controls (Figure 3B). Thus, under the experimental conditions evaluated, PS-MTM treatment resulted in the absence of detectable residual CSFV infectivity in both cell culture-derived virus preparations and clinical samples representing two viral genotypes and markedly different starting infectivity titers.

### 3.2. PS-MTM Treatment Preserved CSFV RNA Detectability by RT-qPCR

The effect of PS-MTM treatment on molecular detection was initially assessed using the Alfort/187 reference strain. Before treatment, the viral preparation exhibited a Ct value of approximately 17.38. Following PS-MTM treatment (dilution 1:2), CSFV RNA remained readily detectable, with a Ct value of approximately 19.13 in the pooled material obtained from the four independently treated preparations. The corresponding 10^−2^ dilution remained RT-qPCR positive, with a Ct value of approximately 26.25. These findings showed that the CSFV RT-qPCR target remained detectable following PS-MTM treatment and subsequent dilution. A similar pattern was observed in clinical samples obtained from pigs experimentally infected with the Catalonia01 strain. Before treatment, serum samples showed Ct values of approximately 17–18, whereas nasal swabs showed Ct values of approximately 21–23. Immediately after PS-MTM treatment, Ct values ranged from approximately 18 to 20 in serum and from 22 to 27 in nasal swabs (dilution 1:2). All Catalonia01-derived samples therefore remained positive by RT-qPCR after treatment (Table 1).

### 3.3. CSFV RNA Remained Detectable During Storage of PS-MTM-Treated Clinical Samples

**Figure 4:**
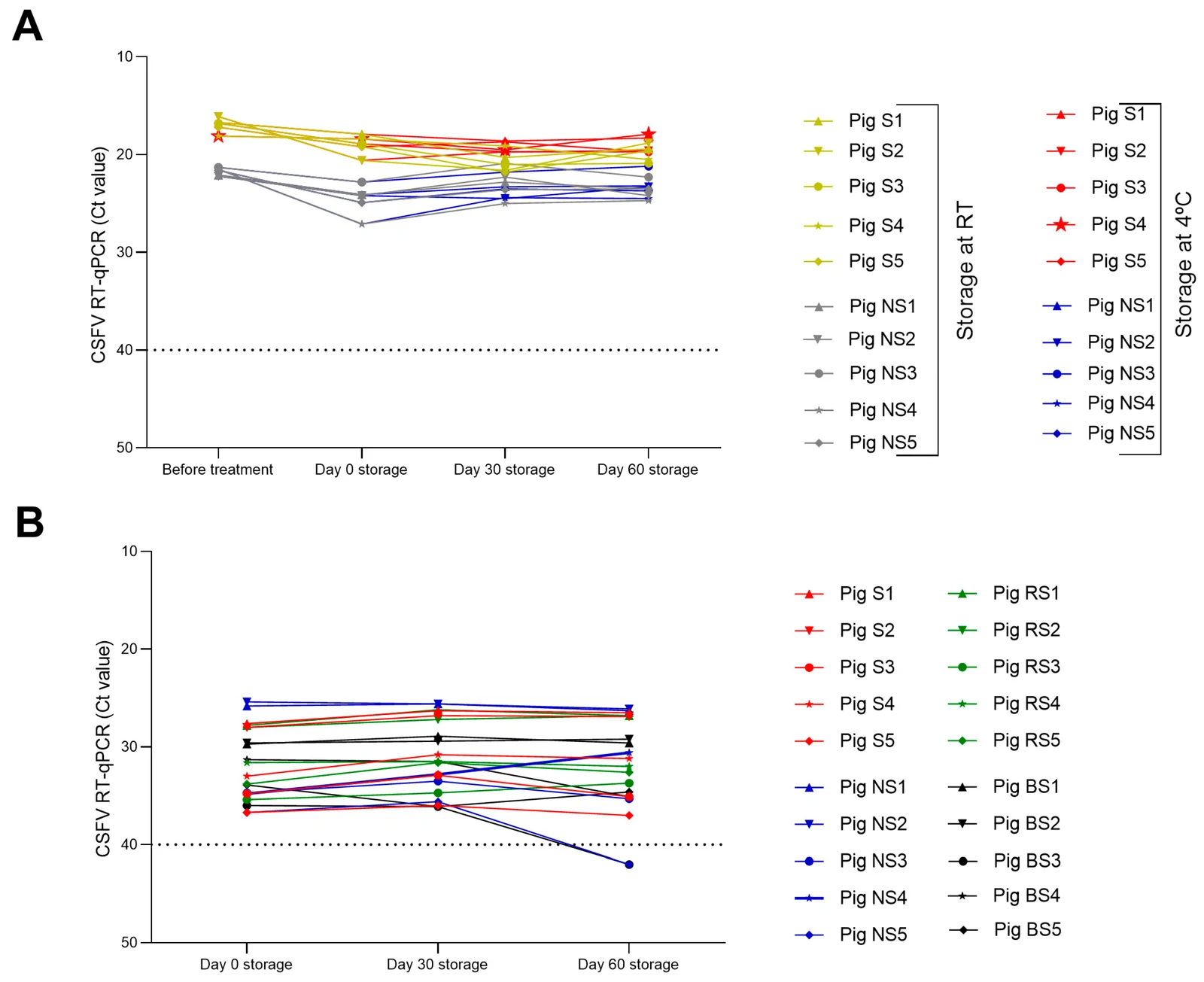
**CSFV RNA detectability in clinical samples following PS-MTM treatment.** (**A**) CSFV RNA detectability in serum and nasal swab samples collected from pigs experimentally infected with the CSFV Catalonia01 strain. Samples were analyzed by RT-qPCR before treatment, immediately after PS-MTM treatment (day 0), and after 30 and 60 days of storage at room temperature or 4 °C. (**B**) CSFV RNA detectability in serum, nasal swab, rectal swab, and buccal swab samples collected from pigs experimentally infected with the highly virulent CSFV Margarita strain. Samples were analyzed by RT-qPCR immediately after PS-MTM treatment (day 0) and after 30 and 60 days of storage at 4 °C. In both panels, the dotted horizontal line indicates the assay positivity threshold (Ct = 40). S, serum; NS, nasal swab; RS, rectal swab; BS, buccal swab; RT, room temperature.

The stability of RT-qPCR detection was evaluated during storage of PS-MTM-treated clinical samples. Serum and nasal swab samples obtained from Catalonia01-infected pigs were stored at either 4 °C or room temperature and analyzed immediately after treatment and after 30 and 60 days. CSFV RNA remained detectable in all evaluated Catalonia01-derived samples throughout the 60-day storage period under both temperature conditions. Individual Ct trajectories showed limited variation over time, with changes generally remaining below two Ct units relative to the post-treatment day 0 measurement (Figure 4A).

Long-term CSFV RNA detectability was also evaluated in serum, nasal, buccal, and rectal swab samples obtained from pigs experimentally infected with the highly virulent CSFV Margarita strain. Samples were stored at 4 °C and analyzed immediately after PS-MTM treatment and after 30 and 60 days of storage. CSFV RNA remained detectable in all evaluated samples at day 30. At day 60, all samples remained RT-qPCR positive except one nasal swab and one buccal swab, which yielded a Ct value above the predefined positivity threshold of 40 and was therefore classified as negative (Figure 4B). Overall, only limited variations in Ct values were observed across sampling times for the remaining samples.

## 4. Discussion

Reliable virus inactivation strategies that preserve nucleic acid integrity are essential for molecular diagnostics of high-consequence animal pathogens. While several approaches have been described for CSFV inactivation [4,5,6,7,8,9,10,11,12], relatively few have been specifically designed to simultaneously eliminate infectivity, preserve viral RNA, and facilitate the safe transport of diagnostic samples. The present results demonstrate that PS-MTM fulfills these requirements under the experimental conditions evaluated, eliminating detectable CSFV infectivity while maintaining viral RNA detectability in both cell culture-derived virus and clinically relevant porcine samples. The present study demonstrates that PS-MTM eliminated detectable residual CSFV infectivity while preserving viral RNA detectability in both cell culture-derived virus and clinically relevant porcine samples. This dual performance is particularly important for molecular diagnostics, where an effective inactivation strategy must simultaneously ensure biosafety and maintain compatibility with downstream nucleic acid detection. The observed results are consistent with the intended mechanism of PS-MTM, a chaotropic transport medium designed to disrupt viral particles, inactivate nucleases, and stabilize nucleic acids. Similar preservation of viral nucleic acids following loss of infectivity has previously been reported for several high-consequence human and animal pathogens [14,16,17]. However, to our knowledge, this is the first peer-reviewed study demonstrating this performance for CSFV in diagnostically relevant porcine samples.

Notably, the absence of detectable residual CSFV infectivity was observed not only in high-titer cell culture-derived Alfort/187 virus but also in diagnostically relevant serum and nasal swab samples collected from pigs experimentally infected with the Catalonia01 strain. The consistency of these results across independent PS-MTM treatments, extensive technical replication, and three consecutive blind passages provides strong experimental support for the robustness of the inactivation protocol under the conditions evaluated. Moreover, the successful inactivation of samples spanning markedly different infectious titers and biological matrices suggests that the performance of PS-MTM is not restricted to a single sample type, reinforcing its potential applicability in routine CSFV molecular diagnostic workflows.

Importantly, the inclusion of diagnostically relevant clinical matrices strengthens the significance of these findings, as the efficacy of chemical inactivation may be influenced by sample composition, organic content, and viral load [8,9,10,11,12,13]. The absence of detectable infectivity in both high-titer serum and lower-titer nasal swab samples further indicates that the inactivation performance of PS-MTM was maintained across clinically relevant matrices spanning markedly different infectious titers. Likewise, the consistent results obtained with the Alfort/187 and Catalonia01 strains, representing CSFV genotypes 1.1 and 2.3, suggest that the observed performance is unlikely to be strain-specific. Nevertheless, additional studies including a broader range of CSFV genotypes, field isolates, and clinical matrices will be necessary to establish the wider applicability of this approach.

The cytotoxicity observed at the 10^−1^ dilution was consistent with the chaotropic composition of PS-MTM and precluded reliable interpretation of virus isolation at this concentration. Consequently, residual infectivity was assessed at the 10^−2^ dilution, representing the highest concentration compatible with accurate evaluation of PK-15 cell monolayers under the experimental conditions used. Although this dilution inevitably defines the analytical sensitivity of the infectivity assay, the extensive technical replication and three consecutive blind passages substantially increased confidence in the absence of detectable residual infectivity under the conditions evaluated.

PS-MTM treatment preserved CSFV RNA detectability by RT-qPCR in both cell culture-derived virus and the clinical samples evaluated. The modest increase in Ct values observed immediately after treatment was consistent with the dilution introduced by the sample-to-reagent ratio and did not indicate substantial loss of RT-qPCR detectability. Furthermore, CSFV RNA remained detectable throughout the storage period under both refrigerated and room-temperature conditions, demonstrating that PS-MTM effectively maintained compatibility with downstream molecular detection across the clinically relevant matrices evaluated. Previous studies have demonstrated that PS-MTM-treated samples are compatible with multiple downstream nucleic acid extraction platforms while preserving nucleic acid integrity for molecular applications, including RT-qPCR and sequencing, across several high-consequence RNA viruses, including members of the family *Flaviviridae*, to which CSFV belongs [14]. Consistent with these findings, viral inactivation occurs immediately upon exposure to PS-MTM and is therefore independent of the subsequent nucleic acid extraction step. Accordingly, the extraction platform used, whether manual or automated, would not be expected to influence the inactivation outcome.

Notably, CSFV RNA remained detectable for up to 60 days in most PS-MTM-treated samples under the storage conditions evaluated, including both refrigerated and room-temperature conditions. These findings demonstrate that PS-MTM preserves RT-qPCR compatibility over prolonged storage, supporting its application for the biosafe transport, storage, and interlaboratory exchange of CSFV-positive specimens without strict dependence on refrigerated shipment.

The absence of detectable residual infectivity together with the preservation of RT-qPCR detectability supports the application of PS-MTM as a practical approach for the biosafe handling, storage, and interlaboratory exchange of CSFV-positive material. These characteristics may facilitate the preparation and distribution of molecular reference materials, the exchange of positive-control samples, and the implementation of proficiency-testing exercises, particularly in settings where access to high-containment facilities is limited. Further evaluation using additional CSFV genotypes, field samples, and diagnostic scenarios will help define the broader applicability of this approach for reference laboratory activities and international surveillance programs.

## 5. Conclusions

This study demonstrates that PS-MTM eliminates detectable CSFV infectivity while preserving viral RNA detectability for downstream RT-qPCR analysis under the experimental conditions evaluated. These findings support the use of PS-MTM as a practical approach for the biosafe handling, storage, and transport of CSFV-positive samples intended for molecular diagnosis. By enabling the safe exchange of non-infectious diagnostic material without compromising RT-qPCR performance, this approach may facilitate interlaboratory collaboration, the distribution of reference materials, and proficiency-testing activities. Its implementation could ultimately contribute to improved diagnostic harmonization and preparedness for the surveillance and control of classical swine fever.

## Figures and Tables

**Table 1:** CSFV RNA detectability before and immediately after PS-MTM treatment.

Sample ID	Ct Before PS-MTM Treatment	Ct Immediately After PS-MTM Treatment
Alfort/187 reference strain	17.38	19.13
Cat01-Pig 1-S	16.78	17.94
Cat01-Pig 2-S	16.14	20.68
Cat01-Pig 3-S	16.84	18.96
Cat01-Pig 4-S	18.14	18.48
Cat01-Pig 5-S	17.20	19.24
Cat01-Pig 1-NS	22.19	24.14
Cat01-Pig 2-NS	22.28	24.29
Cat01-Pig 3-NS	21.34	22.81
Cat01-Pig 4-NS	21.59	27.13
Cat01-Pig 5-NS	21.67	24.96
S, serum; NS, nasal swab		

## Data Availability

The raw data supporting the results presented in this study are available from the corresponding author upon request.

## Abbreviations

The following abbreviations are used in this manuscript:

ATCC	American Type Culture Collection
BS	Buccal swab
BSL-3	Biosafety level 3
CSF	Classical swine fever
CSFV	Classical swine fever virus
Ct	Cycle threshold
dpi	Days post-infection
EU	European Union
EURL	European Union Reference Laboratory
FBS	Fetal bovine serum
NS	Nasal swab
PBS	Phosphate-buffered saline
PCR	Polymerase chain reaction
PK-15	Porcine kidney-15 cell line
PLA	Peroxidase-linked assay
PS-MTM	PrimeStore^®^ Molecular Transport Medium
RNA	Ribonucleic acid
RS	Rectal swab
RT	Room temperature
RT-PCR	Reverse transcription polymerase chain reaction
RT-qPCR	Real-time reverse transcription quantitative polymerase chain reaction
S	Serum
TCID_50_	50% tissue culture infectious dose
WOAH	World Organisation for Animal Health
